# Value of patient-reported outcome measures for evaluating the benefit of speech processor upgrading in patients with cochlear implants

**DOI:** 10.1007/s00106-023-01342-6

**Published:** 2023-09-01

**Authors:** Susen Lailach, Alexander Lenz, Thomas Zahnert, Marcus Neudert

**Affiliations:** Saxonian Cochlear Implant Centre, Department of Otorhinolaryngology, Head and Neck Surgery, Technische Universität Dresden, Fetscherstraße 74, 01307 Dresden, Germany

**Keywords:** Prostheses and implants, Speech audiometry, Hearing tests, Quality improvement, Speech perception

## Abstract

**Background:**

Patients with a cochlear implant (CI) should be evaluated for a new speech processor every 6 years. The aim of this analysis was to assess the subjective and audiological benefit of upgrades.

**Methods:**

Speech understanding and subjective benefit were analyzed in 99 patients with the old and the new speech processor after 4 weeks of wearing. Speech understanding was assessed using the Freiburg monosyllabic test in quiet (FBE) at 65 dB and 80 dB, and the Oldenburg Sentence Test (OLSA) at 65 dB noise with adaptive speech sound level. The Abbreviated Profile of Hearing Aid Benefit (APHAB) was used to assess subjective hearing impairment, and the Audio Processor Satisfaction Questionnaire (APSQ) was used to assess subjective satisfaction.

**Results:**

The speech processor upgrade resulted in a significant improvement of speech understanding in quiet at 65 dB (mean difference 8.9 ± 25.9 percentage points, *p* < 0.001) and 80 dB (mean difference 8.1 ± 29.7 percentage points, *p* < 0.001) and in noise (mean difference 3.2 ± 10.7 dB signal-to-noise ratio [S/N], *p* = 0.006). Using the APHAB, a significant improvement (mean difference 0.07 ± 0.16, *p* < 0.001) in hearing impairment was demonstrated in all listening situations. The APSQ showed significantly higher patient satisfaction with the new speech processor (mean difference 0.42 ± 1.26, *p* = 0.006). A comparative assessment of the benefit based on subjective and speech audiometric results identified a proportion of patients (35–42%) who subjectively benefited from the upgrade but had no measurable benefit based on speech audiometry.

**Conclusion:**

There was a significant improvement in audiologically measurable and subjectively reflected speech understanding and patient satisfaction after the upgrade. In patients with only a small improvement in audiologically measurable speech understanding, the subjective benefit should also be assessed with validated measurement instruments in order to justify an upgrade to the payers in the health sector.

The advancement of externally worn cochlear implant (CI) speech processors is a continuous process. New speech processor developments often focus on ergonomic improvements, such as smaller processor size, improved usability through enhanced controls, or connectivity for external devices [27]. By optimizing speech-coding strategies, signal processing, and microphone technologies, new speech processors can also help to improve auditory performance [4, 9, 21, 24]. This includes, for example, the introduction of new directional microphone options but also possibilities for automatic adaptation to special listening situations or noise reduction [18–20, 25]. Many of these features have been adopted from modern conventional hearing aids. Moreover, the connectivity with external devices, such as phones, tablets, etc., allows hearing-impaired patients to meet everyday communication requirements more easily and better [27]. Once new speech processors are approved, they will become standard processors for future CI patients. However, long-time CI users can also benefit from an upgrade to a new speech processor, as these are almost always compatible with the previous implants [4, 18]. Therefore, patients who are already fitted with a CI can be re-evaluated for a new speech processor every 6 years. Since this upgrade is associated with high costs for the health insurance carrier, it is necessary to prove the additional benefit of an upgrade. The requirements of the medical services of the health insurance companies in this care process are aimed preferably at a documentation of the audiological benefit [16]. However, previous studies have already shown that the subjective benefit of patients in the CI fitting process does not fully correspond with the audiological results [6, 26]. To address this discrepancy, the use of patient-reported outcome measures (PROMs), especially quality-of-life measures, is increasingly required in the CI care process, also in accordance with the new CI guidelines [8]. However, there are no standardized recommendations for the use of PROMs in the process of speech processor upgrade. Especially for patients without audiological benefit after testing a new speech processor, it is necessary to provide more evidence for the documentation of the subjective benefit.

The primary aim of the present study was therefore the standardized documentation of the subjective benefit of speech processor upgrade in experienced CI users after testing a new speech processor over a period of 4 weeks. Furthermore, the audiological benefit of the upgrade should be recorded and compared with the benefit reported by the patients.

## Patients and methods

The clinical study was reviewed and approved by the ethics committee of the TU Dresden (BO-EK-251062020). As part of the routine treatment process, audiological and subjective outcomes with new and old speech processor were analyzed in adults already fitted with a CI for many years in the recording period 2019–2022.

Speech comprehension in quiet and in noise was recorded before the upgrade with the old speech processor (test time 1) and after a 30-day test phase with the new speech processor (test time 2) from the implant companies MED-EL (Innsbruck, Austria), Cochlear (Sydney, Australia) and Advanced Bionics (Valencia, CA, USA). Subjective hearing impairment and satisfaction with the speech processor were also recorded at both test time-points using PROMs.

### Audiological parameters

The speech audiometric tests were performed with the clinical audiometers AT 900 and AT 1000 (Auritec, Hamburg, Germany) in free-field conditions.

To assess speech comprehension in quiet, the Freiburg Monosyllable Test was performed in free field with new and old speech processors at 65 dB and 80 dB. The contralateral side was unaided at the time of testing and was masked depending on the hearing loss. The Oldenburg Sentence Test (OLSA) was also performed with the old and new speech processor without fitting the contralateral side. The measurement was performed adaptively at 65 dB noise and adaptive sound pressure level in the configuration signal and noise from the front (S0N0). The speech reception threshold was determined as the outcome parameter.

### Patient-reported outcome measurements

#### Abbreviated Profile of Hearing Aid Benefit

The Abbreviated Profile of Hearing Aid Benefit (APHAB) was published in 1995 by Cox and Alexander as a further development of the Profile of Hearing Aid Benefit (PHAB) questionnaire and is the most frequently used measurement instrument in conventional hearing aid fitting for the subjective assessment of hearing ability as well as for the evaluation of the benefit of hearing aid fitting [7]. The instrument is available in 18 languages, and the German version has been validated and standardized [15]. Patients are asked to evaluate on a seven-point scale the extent to which they feel impaired by their hearing loss in the situation described. Lower scores are associated with lower subjective impairment. The questionnaire includes three scales to assess hearing in specific listening situations—EC scale (ease of communication; simple listening situation without background noise), BN scale (background noise; listening with background noise), RV scale (reverberation; listening in large rooms with echo or reverberation situations)—and a scale to characterize reactions to environmental sounds, the AV scale (aversiveness of sounds, hearing perception of loud situations).

#### Audio Processor Satisfaction Questionnaire

The Audio Processor Satisfaction Questionnaire (APSQ) was developed and validated to assess satisfaction with the current speech processor in German [5]. The APSQ consists of three subscales (Comfort, Social Life, Usability) made up of five items each, which are answered using a visual analog scale from 0 to 10. The items are taken from the Hearing Implant Sound Quality Index (HISQUI) and the Speech, Spatial and Qualities of Hearing Scale (SSQ; [1, 10]). Higher scores are associated with higher patient satisfaction.

#### Minimal Clinical Reported Difference

The anchor-based Minimal Clinical Reported Difference (MCID) was determined for the assessment of clinically relevant improvement in PROMs [2, 3, 13]. The anchor-based approach to determine the MCID compares the change within the PROM score with an external criterion, a so-called anchor. A questionnaire, a so-called Global Rating of Change (GRC), was used for this purpose, with which the patients evaluated the change in their disease-specific condition following the speech processor upgrade [13]. A 10-point scale was used as the GRC with a range from −5 (*severely worsened*) to 0 (*unchanged*) to +5 (*severely improved*). Results from > −1 to < +1 were considered no change or insignificant change and were excluded from the analysis. Results of ≥ −2 to ≤ −1 or ≥ 1 to ≤ 2 were considered a small change, equivalent to MCID; results from ≥ −3 to ≤ −2 or ≥ 2 to ≤ 3 were considered moderate change, results from ≥ −4 to ≤ −3 or ≥ 3 to ≤ 4 were considered large change, and results from ≥ −5 to ≤ −4 or ≥ 4 to ≤ 5 were considered very large change [13]. To calculate the MCID, we considered the PROM scores of patients who had scores of ≥ −2 to ≤ −1 or ≥ 1 to ≤ 2 in the GRC. The average of the PROM score difference from the measurement before and after speech processor upgrade yielded the MCID.

### Statistical analysis

Statistical analysis was performed using SPSS (IBM® SPSS® software platform, Ehningen, Germany) and OriginPro 2022 (OriginLab, Northampton, MA, USA). In the case of normal distribution, a *t* test was used for statistical comparison of means before and after speech processor upgrade. The significance level was defined as *p* ≤ 0.05. Mean and standard deviation are shown as characteristic values of descriptive statistics.

## Results

Data from 99 patients (55.4% female and 44.6% male) with a mean age of 60.6 ± 16.8 years (range: 17–88 years) were analyzed.

Of these patients, 69 were fitted with a MED-EL implant, 28 with a Cochlear implant, and two with an Advanced Bionics implant (Table 1).

**Table 1:** Fitting situation of the patients before and after upgrade (*n* = 99)

Company	Speech processor before upgrade		Speech processor after upgrade	
Cochlear (*n* = 28)	Freedom	*n* = 1	CP 950 (Kanso)	*n* = 2
	CP 810	*n* = 13	CP 1150 (Kanso 2)	*n* = 3
	CP 910/920	*n* = 13	CP 1000	*n* = 23
	CP 950 (Kanso)	*n* = 1	–	–
MED-EL (*n* = 69)	Duet 2	*n* = 3	Sonnet	*n* = 3
	Opus 2	*n* = 42	Sonnet 2	*n* = 41
	Sonnet	*n* = 11	Rondo 2	*n* = 11
	Rondo	*n* = 13	Rondo 3	*n* = 14
Advanced Bionics (*n* = 2)	Q70	*n* = 2	Q90	*n* = 1
	–	–	M90	*n* = 1

### Speech audiometric results

**Fig. 1:**
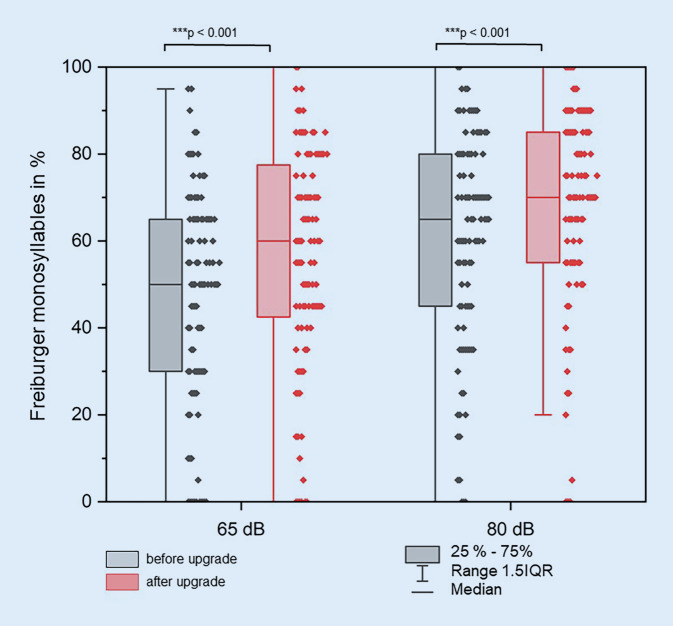
Comparison of Freiburger monosyllables in quiet at 65 dB and 80 dB with old and new speech processor (*n* = 99)

With the new speech processor, the Freiburg Monosyllable Test showed a highly significant improvement in speech comprehension in quiet at 65 dB and 80 dB compared to the previous processor (mean difference 8.9 ± 25.9 percentage points and 8.1 ± 29.7 percentage points, respectively, *p* < 0.001, Fig. 1). The OLSA showed a significant improvement in speech reception threshold from 5.8 ± 13.2 dB S/N to 2.6 ± 7.3 dB S/N (mean difference 3.2 ± 10.7 dB S/N, *p* = 0.006) after speech processor upgrade.

### Patient-reported outcome measures

**Fig. 2:**
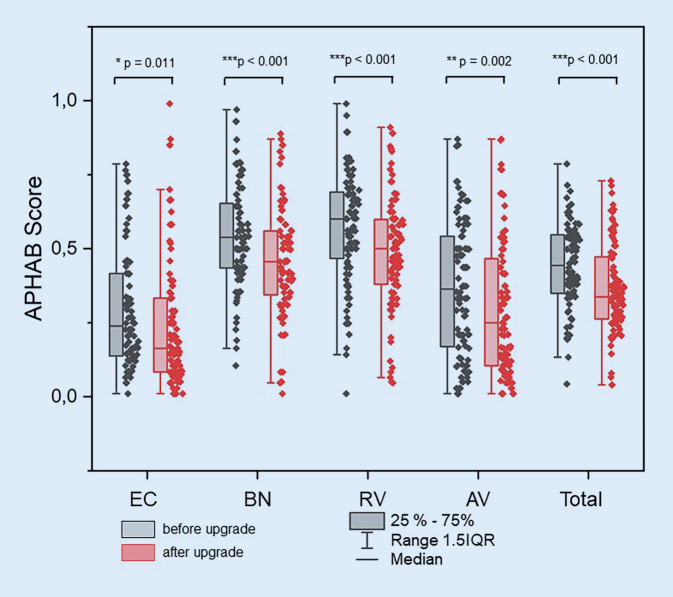
Comparison of subjective hearing impairment based on the Abbreviated Profile of Hearing Aid Benefit (*APHAB*) with old and new speech processor (*n* = 99). *EC* ease of communication, quiet environment; *BN* background noise, *RV* reverberation, *AV* aversiveness of sounds, aversion to loud sounds, *Total* 99< score

**Fig. 3:**
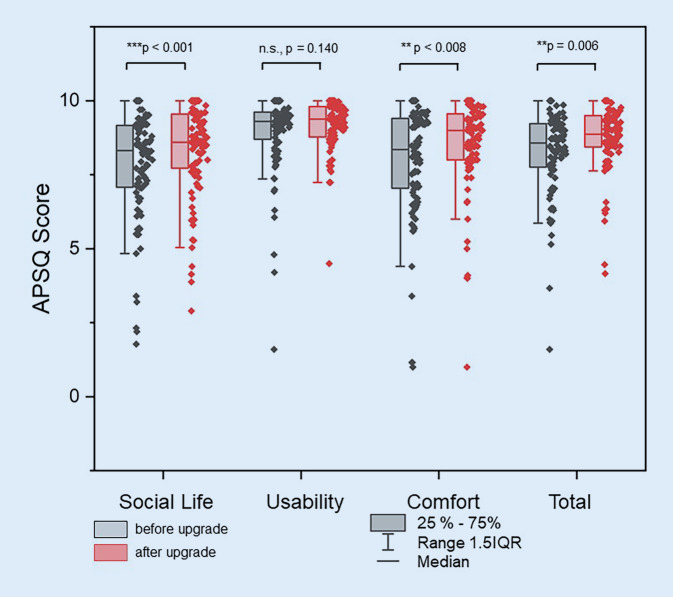
Comparison of satisfaction with the old and new speech processor, recorded with the Audio Processor Satisfaction Questionnaire (*APSQ*; *n* = 99)

When subjective hearing was assessed using the APHAB, a significant (mean difference 0.07 ± 0.16, *p* < 0.001) subjective benefit from speech processor fitting was found for the total score (Fig. 2). In the satisfaction assessment using the APSQ, a significant benefit of care after speech processor upgrade was found for the total score (mean difference: 0.42 ± 1.26, *p* = 0.006) and the subscales Social Life (mean difference: 0.54 ± 1.66, *p* = 0.001) and Comfort (mean difference: 0.57 ± 1.72, *p* = 0.008; Fig. 3).

### Comparison of speech audiometric and subjective evaluation

To assess a speech audiometric improvement due to the new speech processor, a cut-off value of ≥ 20 percentage points in the Freiburg Monosyllable Test at 65 dB or an improvement of 2 dB S/N in the OLSA was set according to the hearing aid guideline [11]. Anchor-based MCID of ≥ 3.8 percentage points was determined for the APHAB total score and 0.74 points for the APSQ total score.

**Fig. 4:**
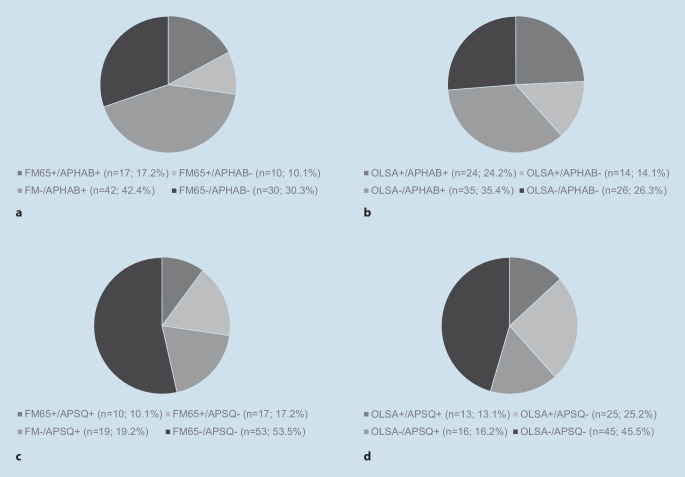
Combined consideration of change in patient-reported outcome measures and speech audiometric results. + improvement, − no improvement, *FM* Freiburger monosyllables, *OLSA* Oldenburger Sentence Test. Defined cut-off values: FM 20%, OLSA 2 dB S/N, Abbreviated Profile of Hearing Aid Benefit (*APHAB*) ≥ 3.8 percentage points, Audio Processor Satisfaction Questionnaire (*APSQ*) 0.74 points

When the Freiburg Monosyllabic Test and the APHAB total score were considered together, 47 patients (47.5%) showed consistent positive or negative results, whereas 52 patients (52.5%) showed incongruent results in this regard. Overall, 42.2% of the patients showed a subjective improvement of hearing in the APHAB, although there was no improvement in the Freiburg Monosyllable Test (Fig. 4a).

When the subjective satisfaction of the upgrade was compared using the APSQ with the results of the Freiburg Monosyllable Test, 63 patients (63.6%) showed consistent positive or negative results. An incongruent evaluation was found for 36 patients (36.4%). Using the APSQ, 19 patients (19.2%) could be identified who subjectively benefited from the upgrade but did not show an improvement in speech comprehension in noise (Fig. 4c).

When the results of the OLSA and APHAB were considered together, 50 patients (50.5%) showed consistent positive or negative results, while 49 patients (49.5%) showed incongruent ratings of the speech processor upgrade (Fig. 4b).

Also, when analyzing the benefit using OLSA and APSQ, a total of 41 patients (41.4%) were identified with different results in speech audiometry and subjective assessment, respectively (Fig. 4d).

## Discussion

The present study was designed to be conducted as part of the routine process of speech processor upgrade. The objective was to evaluate the audiological benefit and the value of integrating PROMs in this process of CI care. The evaluation of individual processor characteristics was not the objective of this study. To evaluate individual properties of a specific processor of an implant company, a controlled study taking into account aspects of hearing biography, age, fitting situation of the contralateral side (e.g., bimodal fitting, single-sided deafness), and year of implantation is necessary. The present study, on the other hand, aims at the actual benefit of the speech processor upgrade for the individual patients in everyday life within the framework of continuous recording including all patients during a fixed period of time on the basis of audiological and subjective evaluation criteria. Due to the continuous recording and the lack of selection criteria, there was a high heterogeneity in the included patients, especially with regard to age, duration of hearing loss, but also implantation period and implant selection. In a similarly heterogeneous group of 233 adults and 261 children/adolescents, speech comprehension before and after speech processor upgrade was compared [23]. There was a significant improvement in speech comprehension of 17.5% in the adult group and 10% in the child/adolescent group.

Previous studies have consistently shown a significant improvement in audiological benefit for each generation of speech processors. Since it is difficult to match patients with respect to hearing parameters and demographic data when comparing different generations of processors, the comparison of different processors was often made during the clinical fitting process, so that the evaluated group of patients was also included as their own control group in nearly all studies. The majority of studies to date have evaluated the benefit of a specific processor from one implant company. For example, clinical studies have confirmed the advantage of the upgrade to the CP810 [18], the C910 [4, 20, 22, 25], the CP1000 [27], the Rondo [17], and the Opus 2 [14, 24]. By contrast, individual studies explicitly evaluate individual technological innovations of a new speech processor in the context of reprovisioning, for example, the effect of specific directional microphone modes [19], signal processing strategies [21], or the speech processor’s connectivity to external devices [27].

Due to the divergent measurement methodology in the individual studies with regard to the speech material used and the test sound pressure levels used, a cross-study evaluation of the benefit of speech processor conversion is hardly possible. Based on the current state of studies, an average improvement of 3–15% is found for monosyllabic test words at 60–65 dB SPL [4, 14, 18, 23, 24]. When measuring with multisyllabic numbers, previous studies have not found a significant improvement related to the upgrade, which at this point can be attributed to a ceiling effect [14]. However, when evaluating the improvement in speech comprehension, it is not only the average increase in speech comprehension that is important, but also the proportion of patients in whom a clinically relevant improvement in speech comprehension is evident from audiological measurements. At this point, a statistically based MCID for the Freiburg Monosyllable Test would be helpful. In routine clinical practice, the difference of 20%, which is taken from conventional hearing aid fitting and which is deposited in the hearing aid guidelines, is typically used as a reference [11]. How many patients actually experience a 20% improvement in speech comprehension has only been sporadically documented in previous studies. In the study by Mosnier et al., for example, 37% of the 35 patients analyzed showed an improvement of at least 20% [18]. Particularly in patients with good speech comprehension, a ceiling effect is to be expected in the measurements in quiet, so that no additional audiological benefit from speech processor upgrade is measurable in these patients. For example, Seebens and Diller showed a smaller improvement (7%) in speech comprehension in patients with already very good speech comprehension compared to the group of all patients analyzed (10%) when evaluating the upgrade to the Opus 2 [24]. Also in patients who did not develop speech comprehension after CI rehabilitation, it is difficult to determine an audiological added value by the upgrade, so that especially for these patients the use of psychometric measurement instruments to assess the subjective benefit is appropriate.

The audiological criteria used by the health insurance companies to assess the need for an upgrade are based on the acceptance criteria for conventional hearing aid fittings. For example, an improvement in speech comprehension of −2 dB S/N in the Oldenburg sentence test is required as a success criterion. By using sentence tests in noise, the aim is to reproduce audioverbal everyday communication. However, the Oldenburg sentence test can only be performed on patients who achieve speech comprehension with the CI. A cross-study evaluation of speech processor upgrade is currently not possible due to divergent measurement methodology and missing reporting standards even in German-speaking countries. There was a tendency, analogous to our study, to show an improvement in speech comprehension in noise [4, 12, 18–20, 23, 24], although it must be taken into account that individual studies explicitly focused on the evaluation of specific noise reduction algorithms or directional microphone modes [12, 19]. In order to capture the advantage of these new developments in each generation of speech processors, it may be helpful to adapt the measurement methodology or set-up for evaluating speech comprehension in noise. For example, the added value of directional microphones only becomes apparent when measured with lateral noise exposure [12].

In Germany, the APHAB questionnaire has been mandatory for years as part of a hearing aid evaluation in accordance with the hearing aid guidelines. With the help of the APHAB, deficits with a therapeutic dimension can be specifically identified, both for the individual patient and for the further development of the hearing aids. The validation of the German APHAB is only available for its use in the fitting process with conventional hearing aids. However, due to its international availability, the APHAB is also increasingly used for the evaluation of CI fitting. The APHAB focuses on the physical domain of hearing loss and is therefore suitable for assessing hearing with old and new speech processors from the patient’s point of view. For all four hearing situations, the patients’ perspective showed a significant improvement in hearing after the speech processor upgrade. Clinical studies on the subjective benefit of the upgrade are currently only sporadically available. Mosnier et al. showed an improvement in hearing with background noise and reverberant environments when converting to the CP810, but no subjective benefit when converting to the CP 910 using the APHAB [18].

In order to cover further aspects of speech processor fitting relevant for the patients, another measuring instrument has to be used. Especially to evaluate wearing comfort and handling of a speech processor, some research groups designed their own questionnaires, partly specified for a specific speech processor, which cannot be recommended for widespread use due to the lack of evidence of validity and reliability. Based on these question lists, a supply advantage with respect to wearing comfort, operation, and hearing impairment with the new processors became apparent [14]. Since these question lists were mostly designed for a specific speech processor, their application when comparing different generations of speech processors is not possible without some doubt involved. This methodological gap could be closed by providing the APSQ developed and validated in German [5]. Although a ceiling effect was found in the previously published study on the validation of the APSQ [5], significant differences in the total score as well as the subdomains between old and new speech processors could be identified in our study. The greatest methodological uncertainty arises in the use of psychometric measurement instruments but also in speech audiometry as a psychoacoustic test procedure due to possible biases, since patients perform the test procedures with the knowledge that they will only receive the new speech processor if it is proven to be superior. However, due to the study methodology and the routine clinical process for evaluating the benefit of speech processor replacement, randomized blinded testing is not possible.

In addition to the standardized assessment of subjective hearing impairment and subjective satisfaction with the respective speech processors, both measurement instruments can also be used to identify patients who do not show any additional audiometric benefit from the speech processor upgrade but who subjectively report a relevant benefit. In our study, a proportion of 35–42% of patients could be identified who did not show any gain in speech comprehension in the audiological examinations, but who subjectively reported a relevant improvement in hearing. In order to be able to justify the upgrade to the health insurance at this point, the inclusion of psychometric measurement instruments in this aspect of CI upgrade should be further supported, since audiological measurements alone do not adequately reflect the benefit of the patients.

As the assessment of specific processor characteristics was not part of the present analysis, it remains unclear which specific characteristics of new generations of speech processors contribute to the subjective benefit of individual patients. Since no control group was chosen, a highly relevant placebo effect cannot be excluded. In follow-up studies with an appropriate control group, the extent of this effect must be identified in order to be able to use the results of the PROMs as a scientific basis for argumentation.

The current requirement in the assessment guidelines of the MDK [16] for a “significant” improvement of speech comprehension in quiet or in noise after the speech processor upgrade for the permission of the new speech processor currently reflects only a very limited view of the payers on this upgrade process. A conclusive sociomedical assessment as a basis for the decision of the healthcare providers requires a comparison of the requested and the assessed need for care, which cannot be determined solely on the basis of audiological data in the case of speech processor upgrade. The combination of different evaluation levels, taking into account validated audiological and psychometric measurement instruments, should in future lend greater weight to applications for the upgrade in the interests of the patient.

## Practical conclusion

- The fitting of a new speech processor in patients with cochlear implants leads to an improvement of speech comprehension in quiet and in noise as well as to an optimization of the subjective hearing impairment and the satisfaction of the patients.
- Patient-reported outcome measures (PROMs) can be used to identify patients who subjectively benefit from a speech processor upgrade but do not show improvement in speech audiometric assessments.
- In order to provide a comprehensive, individualized assessment of benefit, PROMs should be integrated into the routine speech processor fitting process.
- In addition to speech audiometric data, PROMs should provide a basis for argumentation when applying to payers for approval of the upgrade.

## Declarations

The study was reviewed and approved by the Ethics Committee of the Medical Faculty Dresden (BO-EK-251062020). All described investigations in humans were performed with the approval of the responsible ethics committee, in accordance with national law, and in accordance with the Declaration of Helsinki of 1975 (in the current, revised version).

The supplement containing this article is not sponsored by industry.
